# Effects of female obesity on conception, pregnancy and the health of offspring

**DOI:** 10.3389/fendo.2022.949228

**Published:** 2022-08-11

**Authors:** Wei Wei, Xing Zhang, Baotong Zhou, Bo Ge, Jing Tian, Jian Chen

**Affiliations:** ^1^ Key Laboratory of Tumor Immunology and Microenvironmental Regulation of Guangxi, Guilin Medical University, Guilin, China; ^2^ Guangxi Health Commission Key Laboratory of Tumor Immunology and Receptor-Targeted Drug Basic Research, Guilin Medical University, Guilin, China; ^3^ Department of Urology Surgery, The Affiliated Hospital of Guilin Medical University, Guilin Medical University, Guilin, China; ^4^ Department of Urology Surgery, The Second Affiliated Hospital of Guilin Medical University, Guilin Medical University, Guilin, China

**Keywords:** obesity, infertility, embryonic development, offspring, nephropathy, cardiac disease

## Abstract

As we all know, female obesity has become a global epidemic, which is usually accompanied with endocrine and metabolic disorders. Obese women are more likely to experience reproductive problems, including infertility, embryonic developmental defects and abnormality in offspring. Female obesity is a complex multifactorial condition, where there are many mechanisms involved in the effects of overweight and obesity on the development of these reproductive disorders. The insulin resistance, hyperinsulinaemia and hyperandrogenism, lipotoxicity and inflammation are important mechanisms. However, the precise mechanism concerning their correlation is still unclear. Fortunately, weight loss methods have been found to reverse the effects of maternal obesity on the fertility, fetus and offspring.

## Introduction

Overweight and obesity are defined as abnormal or excessive fat accumulation that can damage health. The World Health Organization (WHO) recommends use of BMI to classify overweight and obesity in adults. BMI is defined as weight in kilograms divided by height in meters squared (kg/m^2^). Adults with a BMI of ≥ 25 are regarded as overweight, whereas those with a BMI of ≥ 30 as obese (1). In recent decades, the incidence of obesity has risen at an alarming rate worldwide, and is reaching epidemic levels. In 2015, an estimated 1.9 billion and 609 million adults were respectively suffering from overweight and obese globally, accounting for about 39% of the world’s population, and generally women have higher rates of obesity than men (2). Studies have shown that obesity increases the risk of many chronic disorders, including cardiovascular disease, hypertension, diabetes and even several cancer types (e.g. colon, breast, endometrium cancers) (3). In addition, a growing body of research has revealed that obese women are at a high risk for reproductive health. Their disturbed reproductive health status tend to associate with poor fertilization, abnormal embryo development, poor offspring growth and vulnerability to disease (4–6). Thus, in present study, we provide an overview for current knowledge of the effect of obesity on fertility, pregnancy outcome and health status of offspring.

## Obesity and female infertility

According to the literature, one out of every seven women of childbearing age is infertile in developed countries, and one out of every four women in developing countries. In some parts of the world, such as South Asia, the Middle East and Central Asia, infertility rates even reach 30% (7). Infertility is caused by various reasons, including tubal defects, malformations of the uterus, ovulatory dysfunction, genital infections, endometriosis and endocrine disorders (8). But one of the leading causes of infertility in females nowadays is obesity that is recognized as an independent risk factor for female infertility. The incidence of infertility in overweight women is three times higher than that of normal-weight women (9). Obesity is characterized by abnormal or excessive fat accumulation in women. Excessive fat, especially visceral adipose tissue, can lead to stimulation of the ovaries and adrenal glands, androgen excess, menstrual disorders, and ultimately lead to infertility. So obesity causes infertility in many ways.

Obesity in childhood or adolescence increases the risk of menstrual disorders in women of childbearing age. Obese women appear to have more menstrual irregularities than those of normal weight. 30% to 47% of overweight or obese women have been reported to have menstrual cycle disorders (10). Obesity is a metabolic disease that is usually associated with increased circulating levels of insulin, followed by increased ovarian androgen secretion (11). Next, excessive adipose tissue aromatizes the androgens into estrogen, leading to negative feedback on the hypothalamic-pituitary axis (HPO) and finally decrease in production of gonadotropins (12). Gonadotropins play distinct roles in follicle development, oocyte maturation and corpus luteum formation (13). As a consequence, the lower level of gonadotropins lead to inhibition of ovarian activity, as well as menstrual abnormalities and infertility.

In human pregnancies, the embryo first attaches to the uterine luminal epitheliuml, and then invades into the stroma of the endometrium, where the stromal cells differentiate into decidual cells and provide nutrients to the developing embryo (14). The adipose tissue may affect endometrium functions through the production of many factors such as leptin, free fatty acids (FFA) and cytokines. Studies have shown that endometrial decidualization is impaired in mice with diet-induced obesity (DIO) (15). Compared with normal diet mice, the DIO mice had significant reduction in the number of implantation sites and decreased response of endometrial stromal cells to hormonal stimulation. Similarly, human endometrial stromal cells in obese women experiences reduced ability to undergo normal decidualization, which could inhibit endometrial receptivity. As is known, the optimal endometrial receptivity ensures the successful embryo implantation (16). Therefore, the impaired endometrial receptivity in obese women is responsible for failure embryo implantation and infertility.

Polycystic ovary syndrome (PCOS) is a common endocrine abnormalities of women of reproductive age, and underpinned by insulin resistance and hyperandrogenism. The prevalence of PCOS in obese women is close to 30%, yet obesity is not necessarily the cause of PCOS (17). Actually, consistent data support a bidirectional link between obesity and PCOS. Obesity exacerbates the symptoms of PCOS because it leads to insulin resistance and adipokine release (18). Recent studies have implicated visceral fat as a contributor to insulin resistance by releasing specific adipokine and fatty acid, and thereby contributing to metabolic dysfunction in PCOS. On the other hand, the women with PCOS are more susceptible to weight gain than women without PCOS, which may be mediated by abnormal energy expenditure, excessive androgen secretion, PCOS-related emotional barriers and physical inactivity (19). However, in view that PCOS is a complex multifactorial condition, there is a lack of clear evidence supporting the role of PCOS in weight-gain, not to mention the underlying molecular mechanism.

PCOS is defined by a combination of three major symptoms of hyperandrogenism, ovarian dysfunction and presence of polycystic ovaries (20). The enhanced ovarian androgen production could impair follicular growth by stimulating atresia and apoptosis. The role of androgens in anovulation could be demonstrated by the ovulation restoration in PCOS patients treated with the antiandrogen for six months. During ovulation, the degradation of collagenous tissue in the follicle wall is necessary, in which matrix metalloproteinases (MMP) has a key role. A study in a dehydroepiandrosterone-induced rat model of PCOS demonstrated that MMP2 activity was significantly down-regulated whereas lysyl oxidase (LOX) activity was up-regulated in response to androgens, indicating that androgens could inhibit collagen breakdown and thus cause anovulation in PCOS. Furthermore, not only the number of small antral follicles but production of anti-Müllerian hormone (AMH) by each individual follicle significantly increase in women with POCS, compared with those without PCOS. The increased AMH concentration would lead to more secretion of GnRH by hypothalamic neurons, which would then stimulate luteinizing hormone (LH) production by the anterior pituitary gland and progesterone (rather than estradiol) production by ovary in the end. The premature progesterone rise at the end of the follicular phase seems to advance endometrial maturation and impair endometrial receptivity, leading to embryo-endometrium asynchrony.

## Obesity and embryonic developmental abnormalities

It is reported that one-fifth women start pregnancy as obese, while 20–40% of women gain more weight than recommended during pregnancy. According to the World Health Organization (WHO), reports that the prevalence of obesity in pregnancy varies from 1.8 to 25.3% (21). The maternal obesity poses a threat to the lives of mothers and babies. The fetal risks include preterm birth, macrosomia, congenital abnormalities, preterm births and perinatal death.

The maternal obesity is regarded as a stronger predictor of fetal macrosomia than maternal hyperglycemia (22). Obesity in pregnant women significantly increases the risk of fetal macrosomia, affecting about 20% of newborns. It has been documented that overweight pregnant women have a greater placental weight than those of normal-weight pregnant women (23). The placenta is a combination of a fetal portion (the chorion) and a maternal portion (the decidua), and provides an exchange interface for gas, nutrient and waste products between the mother and the fetus. Macrosomia is typically defined as a birth weight above the 90th percentile for gestational age or >4,000 g. There is a high correlation between the placenta weight and the birth weight (24). The pathophysiology of macrosomia can be explained by the maternal hyperglycemia caused by insulin resistance, leading to elevated placental glucose transport and endogenous fetal insulin secretion. Hence, there is increased utilization of glucose and hyperplastic growth of fetal adipose and protein tissues. However, the relationship between fetal growth restriction and maternal obesity were also demonstrated in obese pregnant women, yet the mechanisms of this relationship are not fully understood. The fetal growth is mainly determined by placental functions, nutrients transportation through the placenta and genetic factors. Therefore, the impaired placental functions by maternal obesity may be associated with the development of fetal growth restriction, and the fetus fails to reach its full growth potential.

Meanwhile, obese pregnant women have a 30% higher risk for congenital anomalies of neural tube, heart and limb than normal-weight pregnant women (25). The precise mechanism by which maternal obesity impacts fetal development is not known. Maternal obesity is known to be related to increased risk for gestational diabetes mellitus. However, Brite et al. reported that no obvious decrease was observed in the risk of congenital heart disease even after adjusting for glucose levels, suggesting that abnormal glucose metabolism could not fully explain the occurrence of congenital anomalies. In addition to glycemic dysregulation, a wide range of metabolic abnormalities are involved in the pathogenesis of obesity. Thus, the multiple adverse metabolic changes in obese pregnant women may contribute to adverse effects in the fetus.

## Obesity and the offspring health

An increasing number of experiments have demonstrated that maternal obesity increases the prevalence of future metabolic dysfunction and malformation in the offspring. Children of obese mothers are more likely to develop obesity, type 2 diabetes, kidney disease, hypertension and cardiovascular disease as adults (26).

It was reported that maternal obesity increased risk of developing congenital kidney defects in the offspring by 22% (27). Moreover, the offspring of obese mothers had worse glomerular damage in comparison with offspring of normal-weight mothers (28). The mechanism of obesity-related nephropathy in offspring induced by maternal obesity is multifactorial. Lipid metabolism disorders have been reported to be associated with the occurrence of kidney disease (29). In the pathogenesis of chronic kidney injury, free fatty acids (FAs) and triglycerides could be freely filtered and reabsorbed by glomerular and tubulointerstitial cells (30). Free fatty acids functions as a link between adipose tissue activity and chronic inflammation, owing to their capability of promoting oxidative stress and inflammation (31). The excess lipid induces the production of reactive oxygen species, pro-fibrotic growth factors and pro-inflammatory cytokines, ultimately developing irreversible tubular damage and tubulointerstitial fibrosis (32). As expected, Yang et al. observed that the inflammation increases cellular uptake of fatty acids, finally triggering glomerular damage in animal experiments (33). Additionally, other metabolic risk factors also may underlie the obesity-related nephropathy such as hypertension, insulin resistance, and dyslipidemia.

Epidemiological studies have revealed a general association between maternal obesity and risk for congenital heart defects in the offspring (e.g. septal defects, conotruncal defects, aortic arch defects). Congenital heart defects, the most common type of malformation, accounts for one-third of all severe malformation (34). There is plenty of evidence showing that increased fat mass is associated with insulin resistance, hyperinsulinemia, lipotoxicity and inflammation, which may adversely impact the embryo development (35). It has also been suggested that maternal obesity may impair the self-renewal in stem cells and induce epigenetic alteration in the embryo, contributing to cardiac malformations (36). Meanwhile, some reported that the offspring of obese women also have an increased risk of being obese. Likewise, increased leptin secretion by excessive adipose tissue leads to insulin resistance and inflammatory response, and thereby directly elicits adverse cardiovascular effects (37). In obese offspring, fatty tissue accumulation increases the stroke volume of the left ventricle, which places an additional burden on the heart. These changes would result in hypertrophy and enlargement of the ventricles, and even predispose patients to the development of heart failure (38).

## Weight loss and the prevention of female reproductive disease

Based on the above, losing weight is important to reduce the risk of obesity-related reproductive dysfunction. In a clinical study involving 170 women undergoing *in vitro* fertilization, women with short-term weight loss have significantly higher production of mid-stage II oocytes than obese women (39). In obese women, reduced-calorie diet and exercise interventions are recognized as an effective way to lose weight, which is associated with improved ovulation and pregnancy rates for women. In a prospective study of 87 obese women, the subjects made changes in diet, exercise and lifestyle for 6 months. Finally, the women who completed the 6 months (total 67) lost an average of 10.2 kg/m^2^. Among them, 90% resumed spontaneous ovulation, 77.6% conceived (32.7% spontaneously), 67% achieved a live birth (40). Conversely, none of these changes occurred in the women who did not complete the treatment. Thus, weight loss can help to improve ovulation, pregnancy and live birth, and thus women with a high BMI should be advised to lose weight prior to conceiving.

## Conclusion

In summary, obesity in women carries the risk of infertility and negative effects on the fetus and offspring. Fortunately, these adverse outcomes can be avoided by moderate weight loss. Meanwhile, for the health of more women, more in-depth research is needed to further understand the relationship between obesity and female reproduction.

## Author contributions

JT and JC for research project with conception, organization, and execution. WW, XZ, BZ, and BG for statistical analysis with design, execution, review, and critique. JT and JC for manuscript preparation with writing of the first draft, review, and critique. All authors contributed to the article and approved the submitted version.

## Funding

This research is respectively supported by the National Natural Science Foundation of China (No.81973574, No.82174082, No.81860443), and the Natural Science Foundation of Guangxi (No.2019GXNSFFA245001). Scientific Research Promotion Project for Young and Middle-aged Faculty and Staff of Guilin Medical University ((No. 2018glmcy033).

## Conflict of interest

The authors declare that the research was conducted in the absence of any commercial or financial relationships that could be construed as a potential conflict of interest.

## Publisher’s note

All claims expressed in this article are solely those of the authors and do not necessarily represent those of their affiliated organizations, or those of the publisher, the editors and the reviewers. Any product that may be evaluated in this article, or claim that may be made by its manufacturer, is not guaranteed or endorsed by the publisher.
